# Non-alcoholic fatty liver disease as a risk factor for female sexual dysfunction in premenopausal women

**DOI:** 10.1371/journal.pone.0182708

**Published:** 2017-08-30

**Authors:** Jae Yeon Lee, Dong Wook Shin, Jeong Won Oh, Won Kim, Sae Kyung Joo, Myung Jae Jeon, Sun Min Kim, Jae Moon Yun, Ki Young Son, Jin Ho Park, Belong Cho, Seung Mi Lee

**Affiliations:** 1 Department of Obstetrics and Gynecology, Seoul National University College of Medicine, Seoul, Korea; 2 Department of Obstetrics and Gynecology, Seoul Metropolitan Government Seoul National University Boramae Medical Center, Seoul, Korea; 3 Department of Family Medicine, Seoul National University College of Medicine, Seoul, Korea; 4 Health Promotion Center, Seoul National University Hospital, Seoul, Korea; 5 Department of Family Medicine, Samsung Medical Center, Seoul, Korea; 6 Department of Internal Medicine, Seoul Metropolitan Government Seoul National University Boramae Medical Center, Seoul, Korea; Institute of Medical Research A Lanari-IDIM, University of Buenos Aires-National Council of Scientific and Technological Research (CONICET), ARGENTINA

## Abstract

**Objective:**

Non-alcoholic fatty liver disease (NAFLD) has become a common and important chronic liver disease worldwide. Previous studies have indicated that NAFLD has an adverse effect on the quality of life, but information is lacking about the impact of NAFLD on female sexual dysfunction. The aim of this study was to determine the association between NAFLD and female sexual dysfunction in premenopausal women.

**Methods:**

This retrospective study consisted of premenopausal women who were sexually active and visited the outpatient clinic for a routine health check-up between January 2010 and December 2011. Based on the examination of the liver ultrasound scan, the study population was divided into 2 groups: cases with NAFLD and normal controls (cases without NAFLD). The female sexual function was compared between the two groups of cases. For the assessment of sexual function, a female sexual function index (FSFI) questionnaire was used.

**Results:**

Four hundred seventy women were included, and the prevalence of NAFLD and female sexual dysfunction were 67/470 (14.3%) and 238/470 (50.6%), respectively. Cases with NAFLD had a lower total FSFI score and higher rate of female sexual dysfunction than the normal control [median score of total FSFI (interquartile range): 24.7 (21.9–27.8) in NAFLD vs. 26.7 (23.7–29.8) in normal control, p<0.005; the female sexual dysfunction: 64.2% in NAFLD vs. 48.4% in normal control, p<0.05]. This difference in female sexual dysfunction between the two groups remained significant after adjustment.

**Conclusion:**

NAFLD is associated with female sexual dysfunction in premenopausal women.

## Introduction

The female sexual dysfunction (FSD) is defined as a persistent and recurrent disorder of sexual desire, arousal, orgasm and pain[1]. FSD is a relatively common disorder, and the prevalence of FSD is reported to be 44–55% in premenopausal Korean women[2, 3]. Female sexual function is associated with multidimensional factors, including anatomical, physiological, psychological and social factors[3–6], and can affect the quality of life and interpersonal relationships[7].

Non-alcoholic fatty liver disease (NAFLD) is characterized by lipid infiltration inside the hepatocyte in the absence of viral hepatitis or excessive alcohol consumption, and is the most prevalent cause of chronic liver disease. The reported prevalence of NAFLD is 20 to 30% in Western countries[8] and 12 to 33% in Korea[6, 9]. NAFLD includes a wide spectrum of liver disorders from simple non-alcoholic fatty liver to non-alcoholic steatohepatitis (NASH) and cirrhosis, which can progress to hepatocellular carcinoma[8]. In recent decades NAFLD was reported to have significant impact on public health, and a body of evidences has suggested the association of NAFLD with type 2 diabetes, obesity and cardiovascular disease[10].

In terms of burden of illness, the impact of NAFLD on the quality of life is an important issue, and previous studies have suggested that patients with NAFLD have lower scores in the quality of life[11, 12]. Although one previous report suggested that NAFLD is a risk factor for sexual dysfunction in a male animal model, there is a paucity of information about the impact of NAFLD on female sexual dysfunction[13].

The objective of this study was to investigate the relationship between NAFLD and female sexual dysfunction in premenopausal women.

## Materials and methods

This was a retrospective cohort study consisting of consecutive premenopausal women who were sexually active and visited the outpatient clinic center for health promotion and optimal aging at Seoul National University Hospital for a health check-up between January 2010 and December 2011. The inclusion criteria included the following: 1) premenopausal women 2) sexual activity at least once in the past 4 weeks, and 3) Completion the questionnaires of female sexual function index. Menopause was defined as amenorrhea at least 12 months without other medical/surgical causes. For efficient and comprehensive care, it is our routine practice to use questionnaires to patients visiting our center, such as demographic factors, socioeconomic status, health behavior (smoking or alcohol consumption), and sexual dysfunction. This study was approved by the Institutional Review Board of Seoul National University Hospital. Informed consent was waived by IRB, because of the nature of the current study (retrospective study).

Based on the examination of a liver ultrasound scan, which was routinely performed during the health check-up, the study population was divided depending on the presence or absence of NAFLD: cases with NAFLD and normal controls (cases without NAFLD). In ultrasound scan, hepatic steatosis was defined as the detection of bright liver echo patterns, as described elsewhere.[14] The liver ultrasound examination was performed by experienced radiologists at outpatient clinic center, who were blinded to the female sexual function questionnaires results. We excluded cases that had a history of malignancy, alcohol intake of >140g/week, positive serologic markers for hepatitis B or C virus and HIV, and use of medications, such as oral contraceptive pills and aspirins, which were associated with NAFLD within the past year[15].

The women were also evaluated with fatty liver index, which is well validated index for fatty liver, with the following formula[16].

$$\text{Fatty liver index}\;=\frac{e^{0.953*\text{log}e(\text{triglycerides})+0.139*\text{BMI}+0.718*\text{log}e(\text{ggt})+0.053*\text{waistcircumference }-\;15.745}}{\left(1+e^{0.953*\text{log}e(\text{triglycerides})+0.139*\text{BMI}+0.718*\text{log}e(\text{ggt})+0.053*\text{waistcircumference }-\;15.745}\right)}\;\times \;100$$

The full scale score of FSFI and the incidence of female sexual dysfunction were compared between subjects with NAFLD and normal controls. Female sexual dysfunction was evaluated with the Korean version of the FSFI (female sexual function index), which is a standardized multidimensional self-reported form containing 19-item questionnaire to assess female sexual function[17]. The FSFI evaluates sexual function during the month prior to a scale measurement in six domains which consist of desire, arousal, lubrication, orgasm, satisfaction, and pain. In each domain, the item was scored with a scale of 1 to 5 or 0 to 5, and the full score was calculated by adding the subdomain scores and multiplying the sum with the domain factor. A total score of below 26.55 was indicated as female sexual dysfunction[18].

Comparison of variables representing the categorical data was performed using the Fisher’s exact test. To identify differences between groups, continuous variables were analyzed using the Mann-Whitney U-test. To test the relationship between FSFI and fatty liver index, Person’s correlation coefficient was used. For multiple analysis, logistic regression analysis was conducted by using the backward stepwise technique, and confounding variables in logistic analysis were chosen according to the result of univariate analysis as a risk factor for FSD (p<0.2)[19]. Statistical analyses were conducted using Statistical Package for the Social Sciences (SPSS) statistics for Windows version 20.0 (IBM Corp., Armonk, NY). For all statistical tests, significance was considered as a p-value < 0.05.

## Results

During the study period, a total of 582 premenopausal sexually active women were approached, and 548 completed the FSFI questionnaire among approached women. After excluding 78 cases (due to the following exclusion criteria: a history of malignancy, alcohol intake of >140g/week, positive serologic markers for hepatitis B or C virus and HIV, or use of medications associated with NAFLD within the past year such as valproate, amiodarone, methotrexate, tamoxifen, or corticosteroids, oral contraceptive pill, and aspirin), a total of 470 women were included in the analysis.

Of the 470 subjects, 67 (14.3%) women were diagnosed with NAFLD based on a liver ultrasound scan. The clinical and general characteristics of the subjects depending on the presence or absence of NAFLD were presented in Table 1. Women with NAFLD had a higher median body mass index (BMI), waist circumference, systolic/diastolic blood pressure, serum levels of C-reactive protein, glucose, hemoglobin A1c (HbA1c), total cholesterol, triglycerides, LDL-cholesterol, alkaline phosphatase (ALP), γ- glutamyl transferase (GGT), and alanine aminotransferase (ALT), and had a higher frequency of hypertension than those without NAFLD. There were no significant difference between the two groups of cases in the median age, median level of alanine aminotransferase (AST), and frequency of marriage, nulliparity, diabetes, current smoking/ alcohol consumption, high education, or high income.

**Table 1:** Characteristics of premenopausal women according to the presence or absence of non-alcoholic fatty liver disease.

Characteristics	Normal control (n = 403)	Cases with NAFLD (n = 67)	p
Age*	39 (34–42)	39 (34–42)	0.631
Body mass index(kg/m^2^)*	20.5 (19.1–22.2)	24.8 (23.0–27.7)	<0.001
Waist circumference (cm) *	74.0 (70.0–78.5)	85.0 (79.0–91.0)	<0.001
Married, n (%)†	333/391(85.2%)	61/66 (92.4%)	0.126
History of child birth, n (%)†	311/400 (77.8%)	56/65 (86.2%)	0.141
Diabetes, n (%)†	3 (0.7%)	2 (3.0%)	0.150
Hypertension, n (%)†	7 (1.7%)	5 (7.5%)	0.018
Systolic blood pressure (mmHg)*	110 (102–119)	122 (114–132)	<0.001
Diastolic blood pressure (mmHg)*	67 (62–73)	74 (67–82)	<0.001
Fasting glucose (mg/dL)*	84 (78–89)	91 (87–101)	<0.001
HbA1c*	5.6 (5.4–5.8)	5.7 (5.5–6.1)	<0.001
Total cholesterol(mg/dL)*	177 (160–197)	190 (168–214)	0.009
Triglyceride (mg/dL)*	66 (54–86)	96 (78–163)	<0.001
HDL cholesterol (mg/dL)*	62 (55–72)	56 (47–59)	<0.001
LDL cholesterol (mg/dL)*	102 (86–122)	121 (105–147)	<0.001
Total bilirubin*	0.9 (0.8–1.1)	0.7 (0.6–0.8)	<0.001
Alkaline phosphatase *	47 (41–54)	53 (45–61)	<0.001
GGT*	14 (12–18)	19 (14–27)	<0.001
AST (U/L)*	18 (16–21)	18 (15–22)	0.726
ALT (U/L)*	13 (11–18)	17 (13–26)	<0.001
Current smoking, n (%)†	25 (6.2%)	7 (10.4%)	0.195
Current alcohol consumption, n (%)†	209 (51.9%)	30 (44.8%)	0.294
High Education (≥ College), n (%)†	304/397 (76.6%)	50/63 (79.4%)	0.748
High Income (≥ 6,000,000KW/month), n (%)†	168/362 (46.4%)	27/58 (46.6%)	1.000
C-reactive protein*	0.01 (0.01–0.04)	0.14 (0.01–0.28)	<0.001
Fatty liver index*	4.51 (2.63–8.46)	24.98 (13.48–48.62)	<0.001

NAFLD, non-alcoholic fatty liver disease; HbA1c, hemoglobin A1c; HDL, high density lipoprotein; LDL, low density lipoprotein; GGT, γ-glutalyltranferase; ALT, alanine aminotransferase; AST, aspartate aminotransferase; KRW, Korean Won.

Fatty liver index = (e ^0.953^*^loge (triglycerides) + 0.139^*^BMI + 0.718^*^loge (ggt) + 0.053^*^waist circumference—15.745^) / (1 + e ^0.953^*^loge (triglycerides) + 0.139^*^BMI + 0.718^*^loge (ggt) + 0.053^*^waist circumference—15.745^) * 100

* Median and interquartile range, compared with Mann-Whitney U test

^†^Compared with Fisher’s exact test

Female sexual dysfunction was reported in 238 (50.6%) individuals out of the total of 470 female subjects, including 195 without NAFLD (control group) and 43 with NAFLD (case group). Table 2 shows the FSFI scores and domain scores of the NAFLD and normal control groups. The FSFI score was lower in cases with NAFLD than in women without NAFLD (median FSFI score (interquartile range): 26.7 (23.7–29.8) for control group and 24.7 (21.9–27.8) for women with NAFLD, p = 0.001), and the median scores for the desire, arousal, orgasm, and satisfaction domains were also lower in cases with NAFLD. Compared to the normal controls, NAFLD women had a significantly higher prevalence of female sexual dysfunction (64.2% vs 48.4%, p = 0.018), and women with NAFLD had a higher incidence of sexual difficulties in the desire, arousal, and satisfaction domains than those without NAFLD. The relationship between NAFLD and FSD remained significant after adjusting for age, waist circumference, parity (history of child birth), hypertension, smoking, and alcohol consumption (Table 3).

**Table 2:** Comparison of female sexual function index scores and frequency of female sexual dysfunction according to the presence or absence of non-alcoholic fatty liver disease.

Characteristics	Normal control (n = 403)	Cases with NAFLD (n = 67)	p
Total FSFI score*	26.7 (23.7–29.8)	24.7 (21.9–27.8)	0.001
Desire*	3.0 (2.4–3.6)	3.0 (2.4–3.6)	0.022
Arousal*	3.9 (3.6–4.8)	3.6 (3.3–4.2)	0.001
Lubrication*	5.4 (4.5–5.7)	5.1 (4.2–5.7)	0.164
Orgasm*	4.4 (4.0–5.2)	4.0 (3.6–4.8)	0.001
Satisfaction*	4.8 (3.6–5.2)	4.4 (3.6–4.8)	0.016
Pain*	5.6 (4.8–6.0)	4.8 (4.0–6.0)	0.121
FSD (FSFI total score ≤ 26.55) †	195 (48.4%)	43 (64.2%)	0.018
Low desire (≤ 2.4) †	137 (34.0%)	33 (49.3%)	0.019
Low arousal (< 3.6) †	79 (19.6%)	23 (34.3%)	0.010
Low lubrication (< 3.6) †	18 (4.5%)	3 (4.5%)	1.000
Low orgasm (< 3.6) †	61 (15.1%)	13 (19.4%)	0.368
Low satisfaction (< 3.6) †	27 (6.7%)	10 (14.9%)	0.028
Sexual pain (< 3.6) †	18 (4.5%)	7 (10.4%)	0.069

FSD, female sexual dysfunction; FSFI, female sexual function index

* Median and interquartile range, compared with Mann-Whitney U test

^†^Compared with Fisher’s exact test

**Table 3:** Relationship of significant variables in female sexual dysfunction by multiple logistic regression analysis.

Variable	Adjust OR	95% CI	P value
Non-alcoholic fatty liver disease	1.889	1.090–3.275	0.023
Age	1.036	1.003–1.070	0.034
Current smoking	2.803	1.221–6.436	0.015

**Fig 1:**
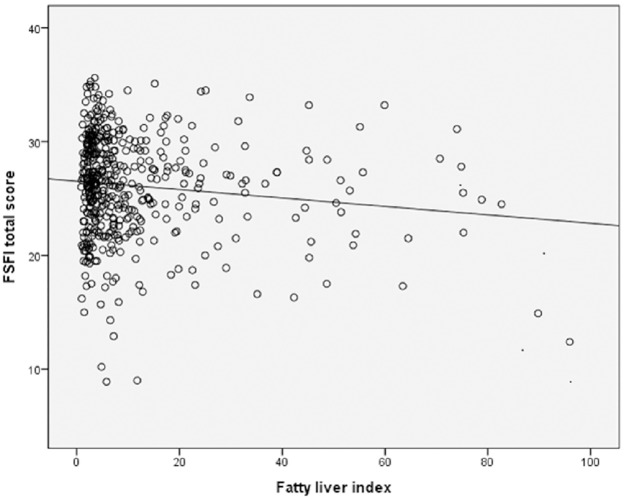
The correlation between fatty liver index and female sexual function index score.

Fig 1 shows the correlation between fatty liver index and FSFI score. Higher fatty liver index was negatively correlated with lower FSFI total score (r = -0.124, p = 0.007).

## Discussion

The primary findings of this study were: 1) The prevalence of NAFLD and sexual dysfunction were 14.3% and 50.6%, respectively; 2) cases with NAFLD had a lower total FSFI score and a higher incidence of female sexual dysfunction than normal control; and 3) the NAFLD was associated with female sexual dysfunction even after adjustment for confounding variables.

Non-alcoholic fatty liver disease (NAFLD) represents a broad clinical spectrum of liver diseases that are defined as lipid accumulation inside hepatocytes [15], ranging from simple non-alcoholic fatty liver to cirrhosis [20]. NAFLD was regarded as “the hepatic manifestation of the metabolic syndrome”, and the recent new paradigm is that NAFLD is a strong determinant for the development of metabolic syndrome[21]. NAFLD has been emerging as a major public health problem in recent decades, and epidemiologic studies have observed the association between NAFLD and type 2 diabetes, obesity and cardiovascular disease[10]. Additionally, it has been shown that NAFLD significantly impairs the quality of life in the general population in terms of lowering physical and mental health scores[11].

A previous report suggested that NAFLD is related to the risk of sexual dysfunction in a male animal model[13]. The authors have suggested that NAFLD can affect gene transcripts in liver homogenates, which are associated with acetylcholine-induced relaxation in the penis[13]. However, we could not find studies specifically designed to assess the association of female sexual function and NAFLD in the literature. One previous study reported no effect of liver disease on female sexual function but this study assessed many etiologies of liver disease, a small percentage of whom had NAFLD[22]. In the current study, we found that NAFLD is associated with FSD. To our knowledge, this study is the largest report so far that evaluated the association between NAFLD and female sexual dysfunction.

Why is NAFLD associated with female sexual dysfunction? Although the mechanisms underlying the correlation between NAFLD and FSD have not been established yet, the findings of the current study can be interpreted by the following putative mechanisms. First, genital engorgement and vascularization may be reduced in patients with NAFLD by increased arterial stiffness and endothelial dysfunction[23, 24]. NAFLD has been known to be associated with arterial stiffness by insulin resistance or increased molecular mediators of atherosclerosis, such as intracellular adhesion molecule and plasminogen activator inhibitor-1 [25, 26]. Endothelial dysfunction in NAFLD can be caused by subclinical inflammation, oxidative stress, procoagulation process with elevate LDL-C and plasminogen activator inhibitor 1 levels, remodeling of elastic properties in the arterial wall, or decreased plasma adiponectin concentrations [23, 24, 26–32].

Second, FSD has been consistently associated with obesity[11]. Thus, adiposity in patients with NAFLD may represent a possible mechanism linking FSD with increased risk of NAFLD. However, in the current study, the association between NAFLD and FSD remained significant even after adjusting for waist circumference. In terms of metabolic syndrome, whether metabolic syndrome is a risk factor for FSD is controversial. In previous studies, the metabolic syndrome or diabetes showed independent role in sexual dysfunction[4, 33–35], but other study reported that the metabolic syndrome was not associated with FSD in middle- to old-aged Korean women[36]. In the current study, the frequency of metabolic syndrome was not different according to the presence or absence of FSD (5.6% in women without FSD vs. 6.7% in women with FSD, p = 0.703).

Third, FSD in NAFLD may be associated with low testosterone levels[37]. Testosterone is an androgenic steroid hormone and binds to sex hormone-binding globulin and albumin, and low level of testosterone can result in sexual dysfunction[38, 39]. The study on sexual function in polycystic ovarian syndrome (PCOS) reported that women with PCOS who have the lowest total serum testosterone concentrations were liable to have the lowest sexual function score [39]. Both the sudden loss of ovarian androgen hormones and the inescapable decline in adrenal sex hormone precursors might be supposed to predispose to the desire and arousal disorders [40]. In the current study, we did not measure the sexual hormone levels in the study population. The association between NAFLD, testosterone levels, and FSD should be investigated in future studies.

This study has several limitations. First, although liver biopsy remains the gold standard for the diagnosis of NAFLD, stage hepatic steatosis, inflammation and severity of fibrosis, NAFLD was defined by the result of abdominal ultrasonography in the current study. Ultrasound diagnosis of hepatic steatosis can result in false positive or negative diagnosis, and the reported sensitivity and specificity of ultrasound for the diagnosis of fatty liver was 92–94% and 80–100%, respectively[41]. Actually, only 11 women had fatty liver index of ≥60 and this suggests that fewer women may really have NAFLD than USG diagnosis. The relatively weak association between fatty liver index and FSFI (r = -0.124 in Fig 1) also suggests the need for further prospective studies with accurate diagnosis. Because of the nature of the current study (retrospective study), whose intention was not initially to assess NAFLD, prospective studies may be needed to confirm the finding of the current study. Second, the study population who visited the outpatient clinic center for health promotion may not be a good representation of premenopausal women. Because of the cost of health check-up (usually between 1,000–3,000 US dollars), the women in this study population may be in higher socioeconomic status, as shown in Table 1. In addition, further studies on the relationship between NAFLD and FSD may be needed in postmenopausal women, in larger cohorts, or in non-Korean population to control for potential confounders not considered in this analysis. In the current study, we have not evaluated the relationship between other hormonal analyses such as estrogen and female sexual dysfunction. Further studies on this hormone are needed to enhance our understanding on the relationship between NAFLD and female sexual dysfunction. In addition, we do not have data on longitudinal changes or durations of NAFLD, because of the nature of the current study (retrospective study). Further studies on follow-up for long-term evaluation of liver disease are needed to enhance our understanding on the relationship between NAFLD and female sexual dysfunction.

In conclusion, NAFLD is associated with sexual dysfunction in premenopausal women.

## Supporting information

S1 File The database of this manuscript. (SAV)
